# Comparison of communication skills between medical students admitted after interviews or on academic merits

**DOI:** 10.1186/1472-6920-12-46

**Published:** 2012-06-22

**Authors:** Marie Dahlin, Stina Söderberg, Ulla Holm, Ingrid Nilsson, Lars-Ove Farnebo

**Affiliations:** 1 Department of Clinical Neuroscience, Centre for Psychiatric Research, St Goran, Karolinska Institutet, Stockholm, Sweden; 2 Stuvsta Primary Care Center, Stockholm County Council, Stockholm, Sweden; 3 Department of Molecular Medicine and Surgery, Karolinska University Hospital, Karolinska Institutet, Stockholm, Sweden

## Abstract

**Background:**

Selection of the best medical students among applicants is debated and many different methods are used. Academic merits predict good academic performance, but students admitted by other pathways need not be less successful. The aim of this study, was to compare communication skills between students admitted to medical school through interviews or on academic merits, respectively.

**Methods:**

A retrospective cohort study. Communication skills at a surgical OSCE in 2008 were assessed independently by two observers using an evaluative rating scale. Correlations, t-tests and multivariate analyses by logistic regressions were employed. Academic merits were defined as upper secondary school grade point average (GPA) or scores from the Swedish Scholastic Assessment Test (SweSAT).

**Results:**

The risk of showing unsatisfactory communicative performance was significantly lower among the students selected by interviews (OR 0.32, CI_95_ 0.12-0.83), compared to those selected on the basis of academic merits. However, there was no significant difference in communication skills scores between the different admission groups; neither did the proportion of high performers differ. No difference in the result of the written examination was seen between groups.

**Conclusions:**

Our results confirm previous experience from many medical schools that students selected in different ways achieve comparable results during the clinical semesters. However, selection through interview seems to reduce the number of students who demonstrate inferior communication skills at 4^th^ year of medical school.

## Background

The selection of students for medical education has been an issue for debate and development since decades. The traditional perspective, that future doctors should be students with high academic performance, has resulted in selection methods based on grade point average (GPA) or high achievement at application tests. Many studies have shown that GPA is a reasonably good predictor of academic performance during the highly science loaded pre-clinical years [1]. Test batteries like the MCAT or BMAT may likewise predict academic performance [2,3], but both these types of selection methods fail to address other crucial requirements of the future professional, such as desirable personal qualities and non-cognitive skills [4,5]. Further, medical school boards as well as health authorities often stress the importance of diversity in the medical student population, which may be counteracted by a biased grading system. In most medical schools the number of applicants by far exceeds the number of places available and in addition, increased rates of applicants with top grades decrease the selective power of GPA. At Karolinska Institutet (KI) the number of applications for 163 places in the fall semester 2010 exceeded 2000.

It has been difficult to find predictors of good clinical performance [1,6]. The role of interviews as an assessment tool for selection is controversial [7]; while some authors have found it useful in predicting clinical performance [8,9] others have questioned its value [10-12]. In part, the difference of outcome may be related to the diversity of both the interview methods – from unstructured to highly structured – and the competence of the interviewers – from laymen with no personality assessment training at all to professionally trained psychologists. One inherent problem is the lack of relevant outcome measures; the basic outcome in any attempt to optimise admissions is a competent clinican.

In Sweden, application for medical school is nationally centralised and there is a quota system where the majority of students are selected from GPA or results of the Swedish Scholastic Assessment Test (SweSAT), which is a generic test, common for application to all higher education. Lottery is applied, since the number of applicants with top grades exceeds the number of available places in the six Swedish medical schools. KI is at present the only Swedish medical school that offers an alternative admittance procedure, primarily based on interviews. This was introduced in 1991 in order to broaden recruitment and with the pronounced objective to identify the most suitable students. With some variation since then, 50-75% of the medical students at KI have been admitted through this procedure.

### Interview admission at KI

To be eligible for the interview application pathway at KI, applicants need a set minimum score on the SweSAT or a set minimum GPA from upper secondary school. A cognitive test (IQ-test) determines which students are invited to the interview, which is then the sole determinant of admission. Interviewers are provided with background information from a written personal statement on life experience and reasons for choosing medicine as a career, used for orientation only. The interview is semi-structured and serves to assess motivation, emotional maturity and stability, cognitive ability, stress tolerance and communication skills; independently rated on a global Likert type scale, range 1-7, where 7 is Excellent. Each category is defined by various subcategories; communication skills are defined by the ability to show empathy, cooperate, make contact and exert leadership. Each applicant is assessed by a psychologist and a medical teacher, respectively, at two separate sessions of 45 minutes each. Rating of the categories is based on information collected during the interview, where the student’s way of handling the interaction is vital. New interviewers are instructed by an interview tutor and all interviewers have yearly training and calibration sessions, to ensure reliability.

Applicants’ scores are ranked; only those with a minimum mean score of 4.5 between raters and with no apparent shortcomings in any of the assessed categories can be admitted, and the final decision is made by an application board.

The effectiveness of this selection procedure has not been thoroughly studied, mostly due to the lack of adequate outcome measures. However, repeated follow-up studies at KI have confirmed that the dropout rate for students selected by interview is lower than for other students [13]. The selection process as such, as well as self-selection may contribute to this finding, i. e. presumptive applicants with unclear motivation do not apply at all or withdraw during the process [14]. Examination scores during the pre-clinical semesters at KI do not differ significantly between students selected by interview or on academic merits [13]. A study of a natural sample of interns in Stockholm found no difference in clinical performance between interns who had been admitted to KI medical school by interview and others [15].

The aim of this study was to compare communication skills and results of the written examination in the surgery course between students selected by interview and those selected on the basis of GPA or SweSAT. Due to the restricted range in both groups, we did not predict a significant difference in the average score outcome between the groups. However, we expected that there would be fewer students rated as unsuitable in terms of serious deficiencies in communication skills in the interview selected group compared with the group selected on academic merits only.

Application for approval was sent to the Ethical Committee of Karolinska Institutet. The Committee saw no ethical conflict and approved the project without the need for formal evaluation.

## Methods

### Setting

Medical school in Sweden covers 11 semesters during 5.5 years; no preparatory courses are given or required. Students pass or fail at all levels and receive no graded marks. Formal communication skills training at KI was at the time of the study given during year 1, intertwined with preclinical courses, and in year 2 during an introductory clinical course. The course in internal medicine is taken during year 3, and in surgery during year 4.

### Students and OSCE

Two cohorts of students taking the course in surgery during the spring and autumn semester 2008 entered the study; in total 241 students. The 18-week course was given at four teaching hospitals, each having approximately 30 students per semester. The course ended with assessment in three parts; a written examination, an Objective Structured Clinical Examination (OSCE) and an oral examination. The written examination and the OSCE were identical at the four teaching hospitals. One of the stations in the OSCE dealt with a simulated patient having abdominal pain (acute pancreatitis due to abuse of alcohol). The student was asked to take a history, perform a physical examination and then inform the patient about possible diagnosis and recommended treatment. This session, of about 13 minutes, was video-recorded. The original recording was copied to two DVDs to allow evaluation by two independent observers.

### Admission groups

Students were admitted through GPA-scores, SweSat results or Interviews. Students who had been denied admittance through the interview procedure could still be accepted in the GPA or SweSAT quota. A small fraction of students were admitted through an interview-based pathway, (similar to the above described) leading to a research preparatory programme, added to the medical school curriculum. The number of students admitted at the autumn semester 2004 and the spring semester 2005, thus scheduled to attend the surgical course at the time of the study, were 264. Of these, 96 (36.4%) were admitted based on GPA-scores or SweSat results and 168 (63.6%) after interviews. During the first semesters a few students were admitted due to distinguished results from the biomedicine course at KI or from other medical schools.

### Evaluation of communication skills and written test

We used a rating scale specifically designed for this study to assess students’ communication skills. We wanted to focus on behaviour dimensions more broadly defined than in checklists, by considering the quality of behaviour and the timing of interactions as more important elements than the frequency of certain behaviours. The rating dimensions thus were evaluative rather than descriptive [16]. Having tested and analysed the recordings of 16 randomly chosen students and adjusted the dimensions and the rating steps according to the findings, we had a final scale consisting of six dimensions. These included the student’s ability to (a) initiate and (b) maintain a relationship with the patient, to (c) give and to (d) gather information, to (e) deal with the patient’s emotions and to (f) organise the consultation.

Each dimension was defined by key behaviours and rated as: inferior (1 p), satisfactory (2 p) and superior (3 p). Maximum points were 18, minimum 6. Cronbach’s α was 0.83.

The raters, three clinical psychologists (IN, UH, SS) and one psychiatrist (MD), all female, initially had 3 training sessions to learn and familiarise themselves with the rating categories and the consultation setting. Raters were blind to the admission group status of the tested students. Since two of the raters are involved as admission interviewers, an initial, independent, sorting was performed to ensure that they would not rate a student they had once interviewed. Subsequently, each recording was randomly assigned to two raters. The inter-rater agreement was 0.69 (Spearman’s rho, p < 0.001). There were small differences in mean score over dimensions between raters (IN 2.34, MD 2.23, UH 2.15, SS 2.18). In order to give the same weight to the result of each rater, the scores were multiplied by a factor (IN 0.85, MD 0.9, UH 0.93, SS 0.92) to obtain the mean 2 for all raters.

The written test consisted of a Modified Essay Question (MEQ) section of three clinical cases, with a total score of 65 and a short-answer questions section, with a total score of 35. The MEQ-section mainly examined clinical reasoning, demanding motivation of all decisions taken regarding the clinical handling of the patient. In total 100 points could be obtained and minimum pass level was 66. No graded marks were set.

### Data and analyses

Gender, age, admission group, communication skills score, and result from the written surgical examination were entered into the database. Communication skills were recorded as a compound score variable, adding scores from the six dimensions. In addition, students could be categorised as “underperformers”, defined as a total score < 10. This level was set in accordance with the generally used pass level at KI medical school of 2/3 of the total score, as it implies a rating of non-satisfactory performance in more than two of the six dimensions. We also tested cut points of 9 and 11. A “superior performer” variable with a total score > 14 was also defined.

GPA was obtained from Statistics Sweden for each selection group, by matching the unique national identification number assigned to all Swedes. GPA-data was only available for the subset of students having finished upper secondary school after 1997, when the grading system in Sweden was changed to the present system where at maximum 20.0 can be obtained. The original GPA when leaving upper secondary school is given, although many students in the GPA group had increased their original GPA to 20.0 after additional studies before admittance to medical school. We were only able to obtain data for subgroups from Statistics Sweden.

Statistics Sweden also calculated Spearman’s correlations between GPA and the communication skills score as well as the results of the written assessment. Descriptive statistics, univariate analyses (other than those performed by Statistics Sweden) and the logistic regression analysis were calculated using software from SPSS (PASWStatistics 18.0). Nonparametric tests were used when data was not normally distributed or of ordinal level, and as group sizes differed. Significance was considered at the 5% level.

## Results

Out of 241 students, 22 who had not been admitted through the two main pathways were excluded (14 exchange students not having Swedish as their mother tongue and 8 students admitted directly from other medical schools or from the biomedicine school at KI). A further 32 students were excluded due to incomplete recordings of the OSCE, in all cases because the recording was not started in time, see Table 1. Valid recordings were obtained for 187 (85.4%) students, 111 women and 76 men.

**Table 1:** Characteristics of sample

		**Excluded group (n = 32)**	**Study group (n = 187)**	**p**
Female		26 (81.3%)	111 (59.4%)	0.018^†^
Male		6	76	
Age, mean (sd)		27.4 (5.1)	26.4 (3.7)	0.321^§^
Admission pathway	Interviews	21 (34.4%)	122 (34.8%)	0.827^†^
	GPA + SweSat	11	65	

^†^Chi^2^-test.

^§^t-test.

Of the tested sample, 134 (71.7%), had been admitted 8 semesters ago, and were thus at pace with the curriculum. The medium length of delay in the remaining group was 1 semester, interquartile range 1-3 semesters.

### GPA-scores

GPA-scores were similar in the interview-research and GPA groups, while lower in the regular interview and the SweSAT groups. The difference between subgroups GPA and regular interview was significant (p < 0.01, t-test), see Table 2.

**Table 2:** GPA scores, results on written examination, communication skills scores and underperformers by admission group

**Admission groups**		**Female**	**Male**	**Age**	**GPA**		**Written exam**	**Communication skills score**	**Under- performers (<10)**
	**n**	**% (n)**	**% (n)**	**Mean (sd)**	**Mean (sd)**	**n^§^**	**Mean (sd)**	**Median (IQR)**	**% (n)**
Interview	112	57.1 (64)	42.9 (48)	26.7 (2.93)	18.02 (1.87)*	96	80.5 (5.7)	11.9 (10.6-13.2)	8.0 (9)
Interview/ research	10	70.0 (7)	30.0 (3)	25.1 (3.70)	19.32 (1.14 )	8	78.3 (6.4)	12.6 (10.4-13.3)	0 (0)
*All interviews*	122	58.2 (71)	41.8 (51)	26.4 (3.0)	-	104	80.3 (5.7)	12.0 (10.6-13.2)	7.4 (9)
SweSAT	14	35.7 (5)	64.3 (9)	30.9 (5.94)	17.78 (2.35 )	6	79.3 (7.0)	11.6 (9.6-12.2)	28.6 (4)
GPA	51	68.6 (35)	31.4 (16)	25.4 (3.58)	19.25 (0.90)*	41	78.8 (6.3)	11.8 (10.2-13.3)	15.7 (8)
*”Traditional”*	65	61.5 (40)	38.5 (25)	26.6 (4.73)	-	47	78.9 (6.4)	11.8 (10.2-13.2)	18.5 (12)

^§^ GPA is given only for the subset of students having finished upper secondary school after 1997, when the grading system in Sweden was changed to the present system where at maximum 20.0 can be obtained. The original GPA when leaving upper secondary school is given, although many students in the GPA group had increased their original GPA to 20.0 after additional studies before admittance to medical school. Data available for subgroups only.

* p < 0.01, for difference in GPA between the Interview and GPA groups (t- test).

### Communication skills at OSCE

There were no significant differences in the distribution of communication skills score between interviewed and not interviewed (Mann-Whitney U, p = 0.340), between the four subgroups (Kruskal-Wallis, p = 0.582), see Table 2. A further analysis regarding possible difference between delayed vs. “at pace” students proved negative (Mann-Whitney U, p = 0.665).

Univariate logistic regression analysis revealed a significantly lower risk of being an underperformer among students admitted after interviews than among those admitted the traditional way, as well as a higher risk among men. These effects were significant also when entered in the same model, while controlling for age (OR 0.32, CI95 0.12-0.83, p = 0.019, Table 3). There was no interaction between admission group and sex. We performed two further analyses; first controlling for examination hospital, which did not affect the model; second, controlling for delay, which did not change the effect of interview admission, but eliminated the effect of sex.

**Table 3:** Effect on underperformance of communication skills by admission after interview vs. “traditional”, sex and age N = 187

	**Unadj OR**	**CI_95_**	**p**	**Adj OR**	**CI_95_**	**p**
Interview (1/0)	0.35	0.14-0.89	0.027	0.32	0.12-0.83	0.019
Male sex	2.66	1.04-6.77	0.041	3.12	1.17-8.30	0.023
Age	0.96	0.84-1.10	0.558	0.93	0.81-1.07	0.338

Logistic regression analysis. Adjusted model: Cox and Schnell R^2^ 0.056, Nagelkerke R^2^ 0.111.

We performed additional analyses to evaluate alternative cut points for underperformers; < 9 (n = 3, 2.5% in interview group; n = 7, 10.8% among non-interviewed) and <11 (n = 38, 31.1% in interview group; n = 28, 40.0% among non-interviewed). The results held for cut point 9, where the effect was more pronounced for interview admission (OR 0.20, CI95 0.05-0.80, p = 0.023), but where neither sex, nor age were significant. For a cut point of 11, which is very close to “satisfactory” performance in all categories (12 points) the effect of interview admission was not significant (OR 0.65, CI95 0.34-1.22, p = 0.175) whereas sex was, (0.39, CI95 0.21-0.74, p = 0.004).

A logistic regression on “superior performers” (communication score above 14), showed no significant effects by interview admission path (OR 0.75, CI_95_ 0.34-1.67), age (OR 1.02, CI_95_ 0.93-1.13) or sex (OR 0.57, CI_95_ 0.24-1.32).

### Written examination

No significant differences were seen in the written examination results between the two selection groups (Mann-Whitney U, p = 0.955), nor by gender (Mann-Whitney U, p = 0.258), Table 2. There was a significant moderate positive correlation between upper secondary school GPA and written examination results (Spearman’s rho 0.29; p < 0.01) and a negative correlation between age and results in the written examination (Spearman’s rho -0.21; p = 0.003).

## Discussion

Our main finding was that the interview procedure seems to reduce the selection of students with inferior communication capability, as assessed at fourth year, in spite of the training in communication skills and other professional behaviour during medical school. However, interview selection did not prove better than traditional selection criteria at distinguishing the students with superior performance.

We did not include analyses of any association between the admission interview ratings and outcome variables, since the variation in admission ratings was minimal in this selected group.

The findings that academic results (= the written test) did not differ between groups and the moderate correlations with GPA, suggest that top GPA from upper secondary school may not be necessary for successful medical studies. This was already shown in a previous evaluation of the different selection pathways at KI [13] and is in line with experience from other medical schools [17].

Studies evaluating selection interviews have used different outcome measures, different time frames and different methods. The value of interview admissions is controversial, and seems to depend on the type of interview and level of experience among interviewers [1]. Still, many medical schools use interviews to assess non-cognitive qualities in applicants. Most studies evaluating interview selection have been performed at institutions where all students are selected by the same standards, thus, within-group correlations are usually the employed analyses [1,18]. Since interviews are not used for all admissions at KI, we were able to compare students that had been admitted as a result of their performance at a selection interview, to those who had not and, in addition, we chose to identify end-points of low and high performers, respectively, which, although it limits variance, yields a measure of certain face-validity.

Many instruments designed to evaluate communication behaviour have been published. Some of these are mainly descriptive in nature [16,19,20], some are evaluative [21] while others combine these two elements [22]. Having carefully analysed video recordings of 16 randomly chosen students we decided to create the evaluation model described in Methods. The internal consistency of the scale was good and the inter-rater reliability was considered acceptable. The cut point chosen for under- and superior performers had not been validated. The cut point for underperforming (<10) was chosen to accord with the general pass level for summative assessments at KI. The choice may indicate some arbitrariness, which is why we tested the effect of two alternative cut points. These analyses showed that also with a more conservative cut point of 9, the effect of interview admission in reducing underperformers was significant, and even more pronounced. With a less conservative cut point of 11, there was no significant effect of interview admission. However, it is doubtful that a cut point that is so close to the norm (median 12, IQR 10.6-13.2 for interviews and 11.8 IQR 10.2-13.2 for academic merits) bears significance in defining underperformance.

There are several limitations to the study. There were significantly more women in the excluded group than in the study group, while admission type and age were similar. They were excluded because the recordings of the OSCE’s were incomplete and thus unratable; there is no reason to assume that failure of recording would be systematically related to the outcome or to gender. Moreover, since we did not find an interaction between gender and admission pathway with respect to inferior communication skills, we do not think that the exclusion affected the results in any systematic way. Also, this was a retrospective study, hence we were not able to examine the attrition rate from different admission pathways. There may thus be a selection bias, where the least able students, both regarding academic results and interpersonal skills, have already left medical school or been delayed in their studies. However, we found no effect of delay among the assessed students, neither on the communication skills score variable, nor for underperforming. Further, only one OSCE station was studied, having included more would have strengthened the reliability of performance rating. However, the other stations were of too short duration or did not include communication skills to a degree sufficient for rating. There were no additional sources available for the assessments of students’ communication skills.

The interview selection process is time-consuming and expensive [23,24] and adequate evaluations of its merits are crucial. Communication skills are a vital part of medical professional competence and this study indicates that the proportion of students with poor communication skills can be reduced with an interview based selection process.

The ranking at the KI interview selection process is designed for selection of the best students, in accordance with the legal frame-work in Sweden, which since 2008 does not allow for universities to employ negative selection, i.e. sorting out the unwanted among applicants, which has otherwise been claimed as a purpose of admission interviews [24]. In spite of this allegation, we found that interview selection rather had the effect of reducing suboptimal performance in such an important area as patient-doctor communication from 18 to 7 per cent. Further studies should evaluate whether the interview process enables a broader recruitment of students with regard to diversity of ethnicity and social economic status.

## Conclusion

The merits of interviews in selection of medical student applicants are debated. This study showed no difference in academic results between students admitted on academic merits or interviews, but suggests that interviews may be successful in reducing the number of students with poor communication capabilities. It is an important task for medical teachers toidentify the underperforming students within the programme, to enable remedial training.

## Competing interests

L-OF was Chairman of the Committee for Selection of Students by Written Test and Interview at Karolinska Institutet during the years 1999-2006. UH and SS have participated as interviewers in this process during many years. MD teaches Psychiatry at Karolinska Institutet medical school.

## Authors’ contributions

L-OF initiated the study and wrote the first draft of the paper. MD conducted the statistical analyses. MD, UH, IN and SS contributed to the study conception and design, elaborated the communication skills scoring and assessed the recordings. All authors contributed to the critical revision of the paper and approved the final version of the manuscript.

## Authors’ information

L-OF is Professor Emeritus of Surgery. Ulla Holm is a clinical psychologist and has published on empathy among physicians. Stina Söderberg and Ingrid Nilsson are clinical psychologists. Marie Dahlin is a psychiatrist and lecturer at KI, her research area is on mental health among medical students.

## Pre-publication history

The pre-publication history for this paper can be accessed here:

http://www.biomedcentral.com/1472-6920/12/46/prepub
